# Transplantation and diabetes (Transdiab): a pilot randomised controlled trial of metformin in impaired glucose tolerance after kidney transplantation

**DOI:** 10.1186/s12882-019-1321-2

**Published:** 2019-04-29

**Authors:** Basil Alnasrallah, Tze Liang Goh, Lai Wan Chan, Paul Manley, Helen Pilmore

**Affiliations:** 1 Department of Nephrology, Auckland City Hospital, Auckland, 1023 New Zealand; 2 Department of Medicine, University of Auckland, Auckland, New Zealand

**Keywords:** Kidney transplantation, Diabetic kidney disease, Post transplant diabetes mellitus, Randomised control trial

## Abstract

**Background:**

Post transplantation diabetes mellitus (PTDM) is a common and serious complication after renal transplantation with significant morbidity and mortality. Metformin has proven benefits in the general population and might be advantageous in the prevention and management of PTDM.

**Methods:**

*Trans*plantation and *Diab*etes (Transdiab) is a single-centre, unblinded, pilot randomised controlled trial assessing the feasibility, tolerability and efficacy of metformin after renal transplantation in patients with impaired glucose tolerance (IGT). Participants had an oral glucose tolerance test (OGTT) in the 4–12 weeks post-transplantation; those with IGT were randomised to standard care or standard care and metformin 500 mg twice daily and followed up for 12 months.

**Results:**

Seventy eight patients had an OGTT over 24 months, 25 of them had IGT, of those, 19 patients were randomised, giving a feasibility of recruitment of 24.4%. Ten patients were randomised to metformin and 9 patients to standard care. Tolerability and efficacy was similar between the 2 groups with no serious adverse events. There was no difference in secondary outcomes relating to the metabolic profile.

**Conclusions:**

The use of metformin post renal transplantation appeared feasible and safe. Larger randomised controlled trials (RCTs) are needed to establish and confirm the efficacy and safety of metformin post renal transplantation**.**

**Trial registration:**

Australian New Zealand Clinical Trials Registry ACTRN12614001171606. Date of registration 7/11/2014.

## Background

End-stage kidney disease (ESKD) is a major public health problem with rising prevalence worldwide. In 2015, there were 4368 people receiving renal replacement therapy for ESKD in New Zealand. Of these, 1694 (39%) had a functioning kidney transplant [1]. Renal transplantation remains the optimal choice for managing ESKD as it offers better survival [2, 3], quality of life [4] and long-term cost [5] compared with dialysis treatment. Despite this, the morbidity and mortality of renal transplant recipients remain considerably higher than that of general population [6], with cardiovascular disease accounting for almost 50% of deaths in these patients [7, 8] and prevalence rates of cardiovascular disease 3–5 times higher than matched general population [9, 10].

An important risk factor for this high mortality and morbidity is post transplantation diabetes mellitus (PTDM). This entity has been recognized for many years with the first cases being described as early as 1964 by Thomas Starzl, but it was in 2003 that consensus guidelines were published setting forth the diagnosis and management recommendations for new onset diabetes after transplantation (NODAT) [11]. More recently the term NODAT was replaced by PTDM as the earlier terminology implies that diabetes prior to transplantation has been adequately excluded which is impractical and often not the case [12].

PTDM is common post transplantation with reported incidence as high 50% [13], however, the true incidence is difficult to determine as there is a wide quoted range which pertains mainly to the heterogeneity of the trials in the literature. Variables such as the transplanted organ, the criteria used for diagnosis, testing times and immunosuppressive regimens all play significant roles in this fact [7, 14].

A combination of traditional and transplant related risk factors are responsible for this risk of PTDM. Of the traditional risk factors, Impaired Glucose Tolerance (IGT), older age, obesity, genetic predisposition, metabolic syndrome, hepatitis C infection and unhealthy life style have all been implicated in the development of PTDM [15–17]. Of the transplant related factors, the lifelong use of immunosuppressive medications plays a dominant role due to their deleterious effects on glucose metabolism, namely, corticosteroids, calcineurin inhibitors (CNIs), and mammalian target of rapamycin (mTOR) inhibitors [17].

IGT post transplantation is also a risk factor for developing PTDM with 15% incidence in 1 year [18] and 27% over 6 years [19]. Furthermore, IGT is associated with increased mortality and overall graft loss [20, 21].

Metformin proved to be an effective therapy to prevent the development of diabetes mellitus in non-transplant patients with impaired glucose metabolism [22]. However, there is lack of evidence for such intervention in transplant patients. Targeting this group of patients will be important to address their PTDM risk.

## Methods

### Research aims

We undertook a pilot study assessing the feasibility, safety, tolerability and efficacy of metformin over 12 months of follow up in patients with IGT diagnosed early after kidney transplantation. The secondary outcomes measured were changes in lipid profile, weight and cardiovascular events.

### Trial registration and ethics approval

The trial has been registered with Australian New Zealand Clinical Trials Registry (ACTRN12614001171606), date of registration 7/11/2014. Ethical approval has been obtained through the Northern B Health and Disability Ethics Committee of the Ministry of Health in New Zealand. Ethics approval number is 14/ STH/129. We have followed CONSORT guidelines for reporting randomized feasibility trials [23].

### Study design

**Fig. 1:**
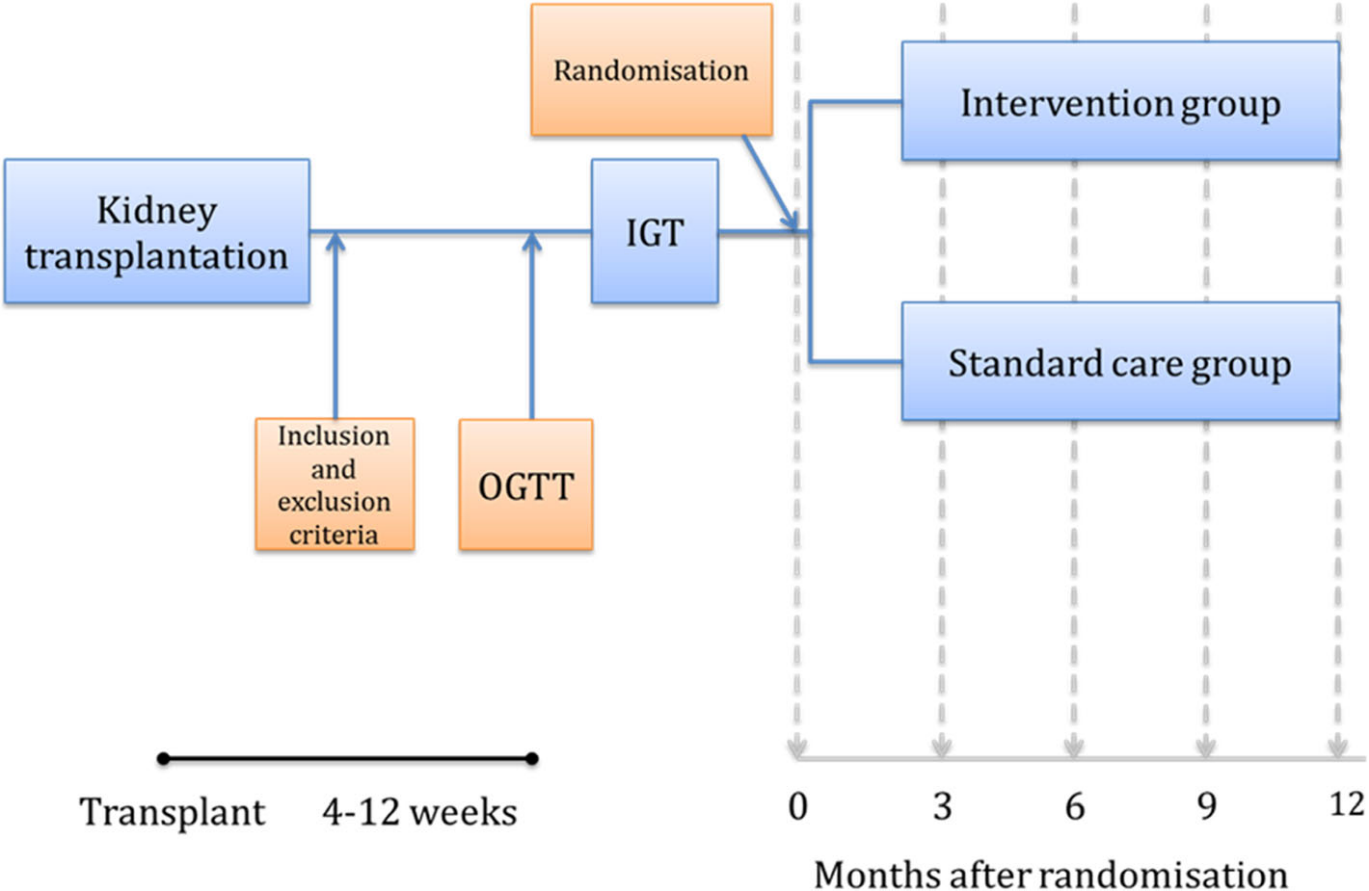
Flow chart of Transdiab trial. IGT, impaired glucose tolerance; OGTT, oral glucose tolerance test

*Trans*plantation and *Diab*etes (Transdiab) is a single-centre, parallel-group, unblinded, randomised controlled trial with two arms: an intervention group and a standard care group. The study protocol in detail has been previously published [24], an outline of the study is shown in Fig. 1.

### Study setting and participants

Adult patients receiving a kidney transplant between 30 November 2014 and 30 November 2016 at Auckland City Hospital, Auckland, New Zealand, were considered for enrollment.

Patients were eligible for **inclusion** if they were 18 years or older, non-diabetic, able to participate in all trial investigations for the 12 month follow-up and able to provide written informed consent.

We **excluded** patients who had one or more the following: diabetes mellitus at the time of transplant (whether on treatment or not), history of antidiabetic therapy, unable to consent, steroid pulse therapy in the 2 weeks prior to OGTT, pregnancy or breast feeding, estimated glomerular filtration rate (eGFR) ≤30 mL/min/1.73 m2 BSA (body surface area) by the Modification of Diet in Renal Disease (MDRD) formula, current substance abuse (including alcohol) and any major illness or comorbidity that may result in death within 12 months.

### Recruitment and randomisation

All potential participants were identified during their admission for the procedure of kidney transplantation. Consented candidates had an OGTT done within 4–12 weeks after the transplantation. Consented patients had their information collected and stored securely, electronically on a password-protected computer and physically in the research office by the research fellow. Enrolling the participants, generating the allocation sequence, and assigning the groups were carried out by the research fellow.

Patients with a 2-h post-load glucose level between 7.8 and 11.1 mmol/L on OGTT (75 g anhydrous glucose dissolved in water) were randomized. Randomisation was done using a computer-generated sequence allocation in closed envelopes in blocks of four in a 1:1 ratio to receive either standard care or standard care plus metformin 500 mg twice daily, both groups were followed up for 1 year.

### Standard care group

Participants in the standard care group received routine post-transplant care directed by the renal transplant team. This included immunosuppressive medications and other treatments as per usual local practice. Standard immunosuppression included basiliximab as induction therapy, mycophenolate mofetil 1 g twice daily, a calcineurin inhibitor (ciclosporin or tacrolimus with dose based on body weight), and steroids (tapering dose over 3 months down to a maintenance of 7.5 mg of prednisone daily).

The lifestyle standard care included advice for regular exercise thrice weekly and a nutritional assessment by a renal dietician. The nutrition care involves guiding patients on the habits of healthy eating and food safety after transplantation.

### Intervention group

Participants randomised to the intervention group received metformin 500 mg twice daily in addition to the standard post-transplant care. The metformin was started at randomisation and continued for 12 months.

## Primary outcomes

### Feasibility of recruitment

Feasibility of recruitment was assessed by examining the percentage of randomised patients of those screened with OGTTs.

### Tolerability of metformin

Tolerability of metformin was evaluated by the gastrointestinal symptom rating scale (GSRS) at 3 and 12 months post randomization. GSRS has been validated to assess symptoms in gastrointestinal disorders [25, 26].

### Efficacy of metformin

Efficacy of metformin was assessed by measuring morning fasting glucose levels and glycated hemoglobin (HbA1c) at 3, 6, 9 and 12 months post randomisation.

## Secondary outcomes

Secondary outcomes included the following: lipid profile at 3, 6, 9 and 12 months; major cardiac events; change in body weight; all adverse events; and proportion of patients who revert to normal glucose metabolism on OGTT at 12 months. The discontinuation or reduction in dose of metformin due to adverse effects was recorded. **Serious adverse events** were defined as events that were fatal or life-threatening, that resulted in clinically significant or persistent disability, that required or prolonged a hospitalization, or that were judged by the investigator to represent a clinically significant harm to the participant that might require medical or surgical intervention to prevent one of the other events listed above [27].

### Statistical analysis

No formal power calculation was undertaken as this was a feasibility study. Patients were analysed based on their original assignments. The feasibility of recruitment was reported as the percentage of patients screened with OGTT who were randomized to either treatment arm. The second primary outcome, tolerability of metformin, was reported as GSRS at 3 and 12 months, compared between the standard and intervention arms using an analysis of covariance (ANCOVA), and adjusted for GSRS at baseline. The third primary outcome, efficacy of metformin, was reported by the levels of HbA1c and morning blood glucose at 3, 6, 9 and 12 months post randomisation and compared between the two groups. This was done using an ANCOVA adjusted for baseline results.

For categorical variables, the comparisons between groups for significant differences were performed using a chi-square test. All statistical analyses were conducted using the statistical software SPSS (version 24.0). The statistical significance level was set at a probability level of < 0.05.

## Results

**Fig. 2:**
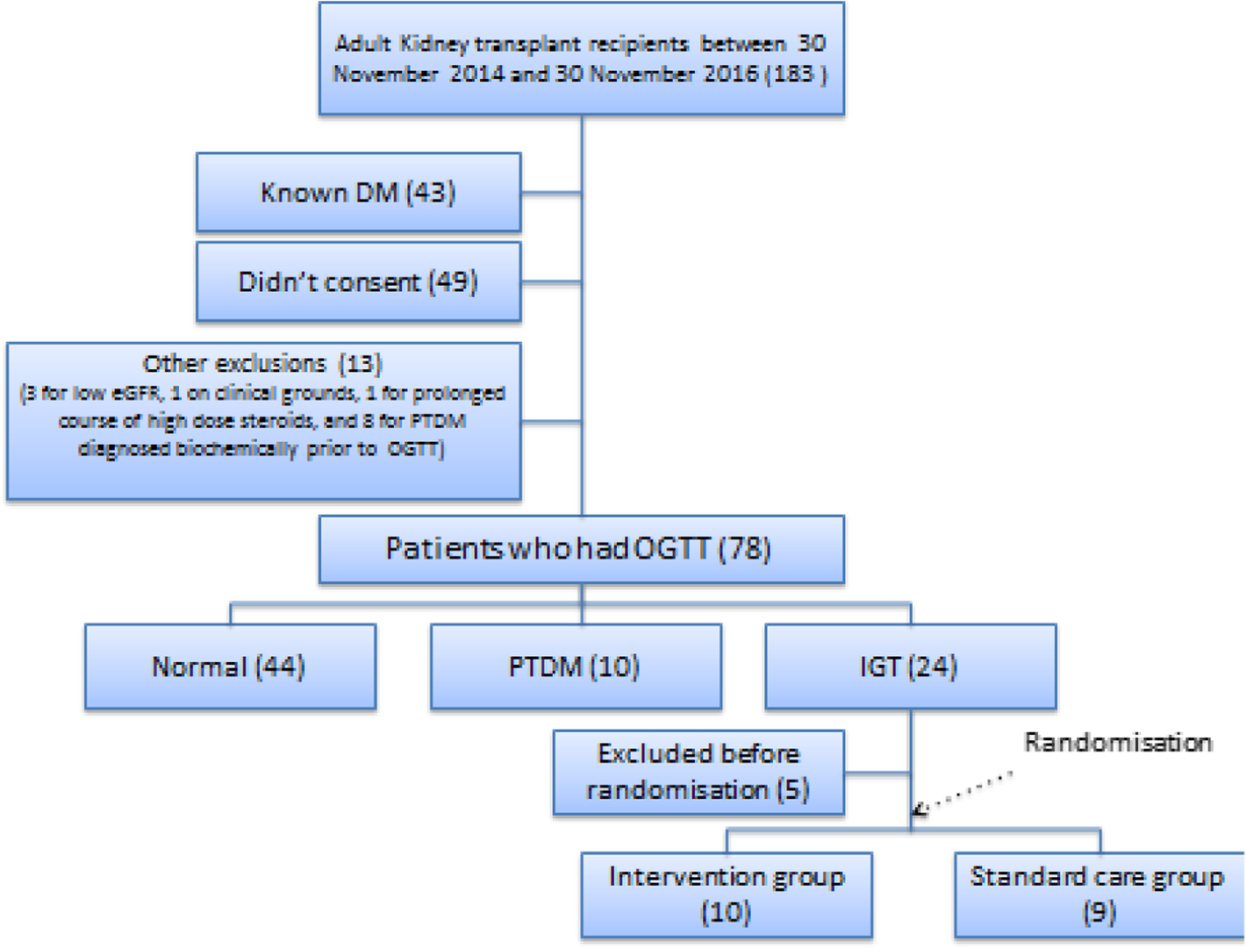
Flow chart of results of recruitment into Transdiab trial. IGT, impaired glucose tolerance; OGTT, oral glucose tolerance test; PTDM, Post transplantation Diabetes Mellitus; DM, Diabetes Mellitus

During the 24-month recruitment period, 183 adult patients received a kidney transplant at Auckland City Hospital, Auckland, New Zealand, of which 105 were excluded largely due to pre-existing diabetes mellitus or unwillingness to consent to the study (Fig. 2). Seventy-eight patients had an OGTT, of which 44 (56.4%) had normal glucose levels, 10 (12.8%) had PTDM on OGTT and 24 (30.8%) had IGT. Of those with IGT, 19 were enrolled and randomized with 10 to the intervention arm and 9 to the control arm. Of the remaining 5 patients, 3 withdrew consent before randomisation and 2 were not randomized for clinical reasons, one for deranged liver function tests (LFTs) and the other for a complicated surgical course and concerns for developing acute kidney injury (AKI).

### Baseline characteristics

The intervention group had more females at 6 patients (60%) compared to the control group with 2 patients (22%). The duration between transplantation and enrolment was longer in the metformin group at 42.3 (standard deviation (SD) 19.8) days compared to 31.8 (SD 6.8) days in the control group, therefore, the steroids tapering dose was higher in the control group at 17.5 (SD 2.6) mg compared to 13.9 (SD 3.3) mg per day in the metformin group. No other significant differences between the 2 groups were identified as per Table 1.

**Table 1:** Baseline characteristics of participants. eGFR, estimated glomerular filtration rate; HbA1c, glycated hemoglobin; OGTT, oral glucose tolerance test, PCR, protein creatinine ratio; TG, triglycerides. NS is not significant when *P*-value is > 0.05.

	Group 1 (Standard care)	Group 2 (Intervention)	*P*-value
Number of patients	9	10	
Females	2 (22%)	6 (60%)	**0.04**
Age (years)	48.5 (SD 11.6)	43.4 (SD 9.3)	NS
Fasting glucose (mmol/L)	5.3 (SD 0.7)	5.4 (SD 0.4)	NS
2 h glucose at OGTT (mmol/L)	9.3 (SD 0.84)	9.8 (SD 0.7)	NS
Body mass index	28.9 (SD 5.6)	26.9 (SD 4.8)	NS
Hba1c (mmol/mol)	35.8 (SD 5.5)	33.8 (SD 3.7)	NS
Creatinine (micromol/L)	136.3 (SD 36.6)	111.4 (SD 37.9)	NS
eGFR in MDRD (ml/min)	55.1 (SD 16.5)	61.4 (SD 20)	NS
GI quality of life	3.1 (SD 3.4)	1.4 (SD 2.5)	NS
Serum cholesterol (mmol/L)	6.2 (SD 1.1)	6.2 (SD 1.2)	NS
Serum TG (mmol/L)	2 (SD 0.9)	2.6 (SD 1)	NS
Systolic BP (mm Hg)	142.3 (SD 16.3)	125.3 (SD 11.4)	NS
Diastolic BP (mm Hg)	85.3 (SD 8.8)	78.9 (SD 7.7)	NS
Duration between transplant and enrolment	31.8 (SD 6.8)	42.3 (SD 19.8)	**0.001**
Urine PCR (mg/mmol)	36 (SD 22.2)	53.8 (SD 35.4)	NS
Steroids dose at randomisation (mg)	17.5 (SD 2.6)	13.9 (SD 3.3)	**0.024**
Number of patients on tacrolimus	3 (33%)	6 (60%)	NS

“Bold entries have clinical significance”

### Primary outcomes

The **feasibility** of recruitment was 24.4% with 19 patients recruited of the 78 who underwent OGTT. The **tolerability** of metformin on GI score was not different between the control and intervention groups at 3 months with scores of 1 (SD 1.6) and 2.3 (SD 2.5) nor at 12 months with scores of 2.3 (SD 4) and 2.9 (SD 1.9), respectively.

**Fig. 3:**
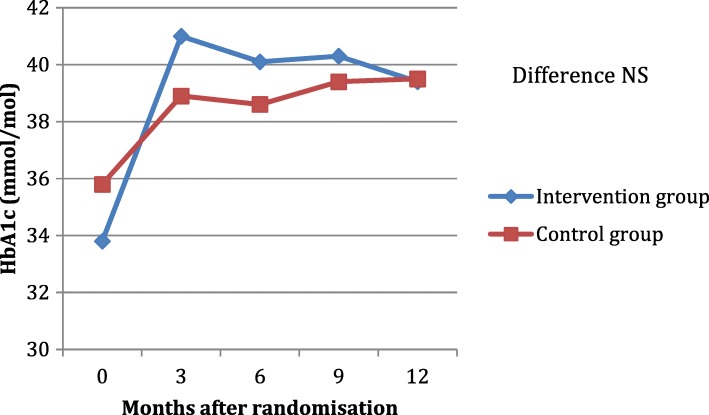
HbA1c levels during follow up in the intervention and control groups. HbA1c, glycated hemoglobin; NS is not significant when *P*-value is > 0.05

**Fig. 4:**
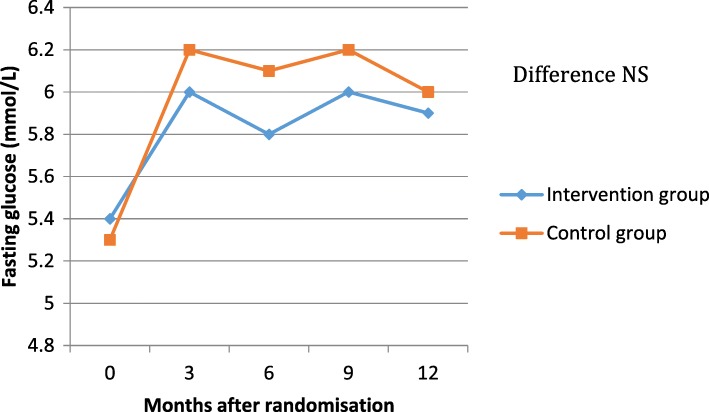
Fasting glucose levels during follow up in the intervention and control groups. NS is not significant when *P*-value is > 0.05

The **efficacy** of metformin on HbA1c and fasting glucose was not different between the 2 groups either at any of the testing points as outlined in Table 2, Figs. 3 and 4.

**Table 2:** Difference between GI scores, fasting glucose and HbA1c between the 2 groups. GI,Gastrointestinal. HbA1c, glycated hemoglobin. NS is not significant when *P*-value is > 0.05.

	Group 1 (Standard care)	Group 2 (Intervention)	*P*-value
GI score at			All NS
3 months	1 (SD 1.6)	2.3 (SD 2.5)	
12 months	2.3 (SD 4.0)	2.9 (SD 1.9)	
Fasting glucose (mmol/L) at			All NS
3 months	6.2 (SD 1)	6 (SD 1.4)	
6 months	6.1 (SD 1)	5.8 (SD 1.1)	
9 months	6.2 (SD 0.7)	6 (SD 1.1)	
12 months	6 (SD 1.1)	5.9 (SD 1.5)	
HbA1c (mmol/mol) at			All NS
3 months	38.9 (SD 4.4)	41 (SD 7.5)	
6 months	38.6 (SD 6.9)	40.1 (SD 6.4)	
9 months	39.4 (SD 7.7)	40.3 (SD 5.3)	
12 months	39.5 (SD 4.6)	39.4 (SD 6.6)

### Secondary outcomes

#### Weight change

Both groups gained weight by the end of 12 months with the intervention group gaining 2.2 kg (SD 5.3) and the control group 6.7 kg (SD 11). However, the difference was not statistically significant (*P*-value 0.12).

#### Lipid profile

There was no significant change in the cholesterol and triglycerides (TG) levels between the 2 groups at 3, 6, 9 and 12 months as per the Table 3. **Cardiac events:** none of the enrolled patients had cardiac events during the follow up.

**Table 3:** Difference between Lipid profile and weight change between the 2 groups. TG, triglycerides; NS is not significant when *P*-value is > 0.05

	Group 1 (Standard care)	Group 2 (Intervention)	*P*-value
Cholesterol (mmol/L) at			All NS
3 months	5.3 (SD 1)	5.3 (SD 1.6)	
6 months	4.9 (SD 0.9)	4.9 (SD 1.1)	
9 months	4.7 (SD 1)	5.5 (SD 1.4)	
12 months	5 (SD 1.2)	5.6 (SD 1.4)	
TG (mmol/L) at			All NS
3 months	2.1 (SD 1)	2 (SD 1)	
6 months	2.1 (SD 0.5)	1.4 (SD 0.7)	
9 months	2 (SD 0.8)	1.9 (SD 1.4)	
12 months	2.3 (SD 1.3)	2.2 (SD 1.7)	
Weight change (kilograms)	+ 6.7 (SD 11)	+ 2.2 (SD 5.3)	NS

### Adverse events

One of the 10 patients discontinued metformin 3 months after enrolment due to gastrointestinal symptoms in the form of indigestion and abdominal pain, which resolved after discontinuation. Another patient had a metallic taste in the mouth 6 months after enrolment, the dose of metformin was halved resulting in resolution of symptoms.

One patient in the control group was started on metformin 500 twice daily 6 months after randomisation by the caring physician due to elevated FBG and HbA1c at 7.9 mmol/l and 54 mmol/mol, respectively.

No patient had a serious adverse drug event and there were no episodes of lactic acidosis.

#### Patients reverting back to normal glucose metabolism on OGTT

60% of patients in the metformin arm (6 patients) and 22% in the control arm (2 patients) returned to a normal OGTT at 12 months, (*p*-value 0.2). One patient in each group developed PTDM and 2 had IGT on the 12-month OGTT in each group.

#### Acute rejection (AR)

Three of the 19 patients had cellular AR (15.8%) during the 12 month follow up, 2 in the metformin group and 1 in the control group; no patients had antibody mediated rejection. All cases of rejection were managed successfully with 3 doses of 500 mg IV methylprednisolone and switching the CNI to tacrolimus if the patient was on cyclosporine.

## Discussion

This is the first randomised controlled trial of metformin in patients after kidney transplantation. The addition of the widely available metformin post transplantation will be simple and practical for high risk individuals if efficacy is confirmed. We found impaired glucose tolerance in over 30% of patients undergoing a glucose tolerance test in the first 3 months after kidney transplantation. Of these 79% were agreeable to participating in a trial of metformin. This indicates that enrolment of patients in studies examining treatments in this sub-group is feasible. Additionally, after excluding patients with diabetes pre-transplant or other exclusion criteria, 61.5% of potential candidates consented to participation in the study. Forty-nine patients refused to consent. This was largely due to reluctance of patients to take extra medications and the fear of side effects.

Most importantly, there were no significant safety concerns with metformin use early post transplantation in this small group of patients. There were no admissions with lactic acidosis and no significant difference in GI symptoms with the scoring system used. The available safety data for the use of metformin in renal transplant patients is limited. A previous retrospective review by Stephens and colleagues showed that metformin has been used by many renal transplant patients in the United States with no evidence of worse patient or allograft outcomes [28]. Also, a small retrospective study in 2008 reported the safety of metformin in renal transplant patients with PTDM and pre-existing diabetes mellitus [29].

Metformin has been available for many years and has many favourable effects which can be of particular benefit to transplant recipients, namely enhancing glucose metabolism, limiting unwanted weight gain, cardiovascular protection, improving metabolic profile, and anti-neoplastic activity [30]. However, a major deterrent for its use in renal transplant patients has been the safety concern, with physicians fearful of precipitating a dangerous lactic acidosis. Metformin is exclusively renally excreted with a clearance correlating approximately linearly with glomerular filtration rate (GFR) [31].

Renal transplant recipients commonly have abnormal eGFRs with frequent fluctuations in renal function, potentially leading to accumulation of metformin and increasing the risk of lactic acidosis. However, the attitude towards the use of metformin in patients with renal impairment has changed in recent years, as has the perceived risk of lactic acidosis in those patients. Recent large reviews have provided reassurance of the safety of metformin in patients with mild-moderate renal impairment (estimated glomerular filtration rates, 30–60 mL/min per 1.73 m2), with no significant difference in the rates of lactic acidosis between those treated with or without metformin [32, 33].

Subsequently, more than one regulatory authority has changed its recommendations allowing for the use of metformin in eGFR less than 60 ml/min and as low as 30 ml/min [34–36].

In our study, the enrolment was 4 weeks or more after transplant, this allowed for the renal function to stabilize. We also excluded those with eGFRs <30mls/min, and educated all enrolled patients about metformin and its potential side effects with action plans when they have health concerns. This allowed for safe application of this intervention, which can be further applied in larger future RCTs.

The other primary outcome, efficacy of metformin, was not statistically different between the 2 groups at any of the designated time points. It would have been challenging to detect a difference with the small sample size. There were no differences in the secondary outcomes between the 2 groups either, and although there was a trend to less weight gain and more patients reverting to normal OGTT at 12 months in the metformin group, this was not statistically significant. Weight gain was noted in both groups, this is a common finding after renal transplantation which can be attributed to a few factors, mainly the use of immunosuppressive medications (such as corticosteroids), improvement in well-being and the relaxation of dietary restrictions [37, 38].

PTDM is associated with increased rates of cardiovascular disease, cardiovascular death and overall mortality [7, 39, 40]. PTDM is also associated with increased overall graft failure [20, 41].

Therefore, finding effective ways to manage PTDM or even better preventing it will help to address those risks. With the limited evidence on the use of Metformin in kidney transplant patients, it is only possible to use data from the general population to estimate the potential benefit of metformin on glucose metabolism in at risk individuals. The Diabetes Prevention Program Research Group found that metformin reduced the incidence of diabetes in patients with elevated fasting glucose and/or impaired glucose tolerance by 31% over an average follow up of 2.8 years compared to placebo, with a number needed to treat (NNT) of 13.9 to prevent one case over 3 years [22]. Also, a meta-analysis reported a similar benefit of metformin with a 40% reduction in the incidence of diabetes mellitus in individuals at high risk over 1.8 years with NNT of 17 [42].

The pathophysiology of the impairment in glucose metabolism appears to be different in PTDM to that in type 2 diabetes mellitus where metformin has been widely used [43, 44]. Therefore, the benefits of metformin on glucose metabolism can’t be automatically extrapolated and need to be tested in the transplant recipients. The impairment in PTDM is related to a combination of β-cell dysfunction and insulin resistance [43–45]. This is related primarily to the diabetogenic effects of immunosuppressive medications, as CNIs, which are the cornerstone of contemporary transplant immunosuppressive therapy lead to beta-cell dysfunction and reduction of insulin secretion [46]. Other immunosuppressives lead to different effects, with steroids inducing peripheral insulin resistance and impairing glucose uptake, the exact diabetogenic mechanism of mTOR inhibitors is still uncertain [46].

The main limitation of our study is the small number of patients enrolled. This was expected for a pilot study carried out in a single transplant center, this limited the ability to confidently adjust for some known risk factors for PTDM like tacrolimus use, obesity and older age. Secondly, the dose of metformin was conservative in this trial at 1 g per day, as the maximal dose is 3 g per day. As our study is the first to use metformin prospectively in this group of patients, a conservative dose was chosen to encourage participation by patients and physicians and to gather initial safety data before advocating for higher doses in future studies. Using a higher dose of metformin is likely to result in more profound therapeutic effects, which can be entertained in future RCTs.

For future studies, intensive dietary regimens, changing immunosuppression when feasible and using pharmacological therapies are all interventions that could be implemented. Multicenter collaboration should be considered to ensure enrolling adequate number of patients where further stratification can be done, i.e. according to renal function, higher metformin doses, and adjusting for known risk factors. i.e. tacrolimus use. Using OGTT at 1 year as an end point for efficacy would be reasonable as it remains the gold standard for PTDM diagnosis [12], also because the overall rate of PTDM remains largely unchanged beyond 1 year post transplantation [47].

## Conclusions

We have demonstrated reasonable feasibility in a trial using metformin in patients with impaired glucose tolerance after kidney transplantation. In this pilot study the use of metformin in renal transplant recipients with IGT appeared safe and had good tolerability with no serious adverse events, this should help planning future larger RCTs. The efficacy of the use of metformin can be further assessed in studies with adequate number of patients to address the pressing issues related to the prevention and management of PTDM.

## Abbreviations

AKI: Acute kidney injury
ANCOVA: Analysis of covariance
AR: Acute rejection
BSA: Body surface area
CNIs: Calcineurin inhibitors
eGFR: estimated glomerular filtration rate
ESKD: End stage kidney disease
FBG: Fasting blood glucose
GSRS: Gastrointestinal symptom rating scale
HbA1c: Glycated hemoglobin
IGT: Impaired glucose tolerance
LFTs: Liver function tests
MALA: Metformin associated lactic acidosis
MDRD: Modification of diet in renal disease
mTOR: Mammalian target of rapamycin
NNT: Number needed to treat
NODAT: New onset diabetes after transplantation
OGTT: Oral glucose tolerance test
PCR: Protein creatinine ratio
PTDM: Post transplantation diabetes mellitus
RCT: Randomised controlled trial
SD: Standard deviation
TG: Triglycerides
